# Intraoperative radiotherapy for breast cancer treatment efficiently targets the tumor bed preventing breast adipose stromal cell outgrowth

**DOI:** 10.1007/s00066-020-01586-z

**Published:** 2020-02-06

**Authors:** Stefanie Uhlig, Anne Wuhrer, Sebastian Berlit, Benjamin Tuschy, Marc Sütterlin, Karen Bieback

**Affiliations:** 1 Institute of Transfusion Medicine and Immunology, Medical Faculty Mannheim, German Red Cross Blood Donor Services, Heidelberg University, Friedrich-Ebert Str. 107, 68167 Mannheim, Germany; 2 FlowCore Mannheim, Medical Faculty Mannheim, Heidelberg University, Ludolf-Krehl Str. 13–17, 68167 Mannheim, Germany; 3 Department of Obstetrics and Gynecology, University Medical Center Mannheim, Heidelberg University, Theodor-Kutzer-Ufer 1–3, 68167 Mannheim, Germany

**Keywords:** Intraoperative radiotherapy, Mesenchymal stromal cells, Adipose stromal cells, Tumor bed, Breast cancer

## Abstract

**Objectives:**

Mesenchymal stromal cells (MSC) in bone marrow have been shown to be radioresistant, which is related to pronounced DNA repair mechanisms. Intraoperative radiotherapy (IORT) during breast-conserving surgery for early breast cancer is an innovative technique applying low energy x‑ray to the tumor bed immediately after removal of the tumor. IORT is considered to reduce the risk of local tumor recurrence by directly targeting cells of the tumor bed and altering the local microenvironment. Aim of this study was to investigate whether IORT affects the outgrowth potential of breast adipose tissue-derived MSC (bASC) as part of the tumor bed.

**Materials and methods:**

After surgical tumor resection, biopsies of the tumor bed were taken before (pre IORT) and after IORT (post IORT) and processed applying well-established protocols for ASC isolation and characterization.

**Results:**

In all, 95% of pre IORT tumor bed samples yielded persistently outgrowing bASC with typical ASC characteristics: fibroblastoid morphology, proliferation, adipogenic and osteogenic differentiation and ASC surface marker expression. However, none of the post IORT samples yielded persistent outgrowth of bASC.

**Conclusions:**

After breast-conserving surgery, approximately 90% of local recurrences emerge in close proximity to the initial tumor bed, potentially reflecting a significant contribution of the tumor bed to relapse. Our data show that IORT, besides the proven effect on breast cancer cells, efficiently modifies the tumor environment by having an impact on tumor bed bASC. This effect on tumor bed stromal cells might contribute to reduce the risk of tumor relapse and metastases.

**Electronic supplementary material:**

The online version of this article (10.1007/s00066-020-01586-z) contains supplementary material, which is available to authorized users.

## Introduction

Breast cancer is the most frequent malignant tumor in women. Beside surgical, chemotherapeutic and receptor-targeted therapy, local radiotherapy completes the mainstays of treatment. While postoperative whole breast radiotherapy remains standard in patients undergoing breast-conserving surgery, intraoperative radiotherapy (IORT) is increasingly implemented in clinical routine. This risk-adapted concept uses low energy x‑rays applied during surgery directly after excision of the tumor [1]. The idea of using local radiotherapy in general is to eliminate potentially remaining tumor cells in the tumor bed after surgery, considered as a major source for relapse [2]. With irradiation taking place in the wound cavity directly after having removed the tumor, the risk of local or temporal miss is hypothetically nonexistent.

As supported by the *seed and soil* theory, the wound healing process after surgery is likely to provide favorable growth conditions not only for the healthy tissue, but also for residual tumor clusters [3]. Subsequently, a modification of the tumor bed stroma and its micromilieu as potentially provoked by IORT could result in a reduction of the risk of local recurrence. Furthermore, the fact that local control is correlated with an improvement in overall survival in an oncological disease with early metastatic spread implies that systemic progress might be substantially affected by mechanisms in the tumor bed [4]. IORT could provide a saturation of the DNA repair system eventually leading to increased genomic instability and thus inactivation of tumor cells [2]. Furthermore, immediate irradiation after excision of the primary tumor could prevent the proliferation and division of residual malignant cells during wound healing [2]. In the scope of breast cancer therapy, the impact of IORT on the tumor bed stroma under in vivo conditions is scarcely investigated, mainly focusing on the wound fluid and not on the cellular part of the tumor bed tissue [5, 6].

Mesenchymal stromal cells (MSC), as a potential part of the tumor bed stroma, comprise a heterogeneous population of multipotent stem/stromal cells that can be isolated from a variety of different tissues including adipose tissue (adipose stromal cells, ASCs) [7]. Due to their regenerative potential, MSCs are considered as promising candidates for diverse clinical applications in cell and gene therapy. In this respect, the fate of MSCs under the influence of ionizing radiation became of particular interest. MSCs have been ascribed with evincing radioprotective and regenerative features in tissues exposed to ionizing radiation, even in patients [8, 9]. Yet, what presents itself as a benefit on the one hand could be considered as a drawback for the oncological outcome, since these protective effects could not only support normal tissue but also tumor cells treated with radiotherapy [9].

In allogeneic bone marrow transplant setting, stromal cells remain host-derived irrespective of the condition regime intensity [10]. This suggests relative radio- and chemoresistance. In fact, ex vivo cultured MSC/ASC are resistant to radiation withstanding even high radiation doses [11].

The aim of this work was to analyze whether IORT affects the outgrowth potential of bASC, indicative for an effect on the tumor bed stroma. Biopsies of breast adipose tissue were harvested in patients with IORT before and after IORT and in control patients without IORT. Outgrowing cells were characterized against MSC criteria.

## Materials and methods

### Patients and intraoperative radiotherapy

A total of 20 breast cancer patients undergoing breast-conserving surgery with (study collective) and 21 without (control collective) IORT were recruited after written informed consent was obtained. All procedures performed in studies involving human participants were in accordance with the ethical standards of the institutional and/or national research committee (No. 2013-589N-MA, Mannheim Ethics committee II) and with the 1964 Helsinki declaration and its later amendments or comparable ethical standards. In women of the study collective, tumor bed biopsies were taken before (pre) and after (post) IORT.

IORT was performed according to the TARGIT‑A protocol [4]: The Intrabeam® system (Carl-Zeiss Meditec AG, Oberkochen, Germany) was used for intraoperative irradiation (50 KeV x‑ray). After excision of the tumor and pathoanatomical confirmation of free margins via frozen section, the spherical Intrabeam applicator was adjusted in the wound cavity. Irradiation was accomplished with a dosage of 20 Gy. In patients undergoing breast-conserving surgery without IORT, biopsies of the tumor bed were taken pre and post sentinel node biopsy (SNB) to ensure a comparable time interval of approximately 30 min between both biopsies. Biopsies were taken using conventional scissors. Of course, patients of the control collective also underwent irradiation but in contrast to the study collective solely 3 to 4 weeks after surgery via conventional whole breast radiotherapy.

Histological subtypes and molecular phenotype of were comparable for IORT and controls (not shown).

### ASC isolation and characterization

Twenty-four hours after biopsy, breast ASC (bASC) were isolated using an established method [7]. Briefly, tissue was weighed and then cut into small pieces. The tissue pieces were digested in 10 ml 0.075% collagenase I (Sigma Aldrich) in prewarmed DMEM (Pan Biotech) for 30–45 min at 37 °C. 20 ml of prewarmed DMEM were added to dilute collagenase and then the cell suspension filtered through a 100 µm cell strainer (Falcon). The cell suspension was then centrifuged (1200 ×g, 10 min, RT) and then optionally treated with erythrocyte lysis buffer (1 ×, 10 min, centrifugation 1200 ×g, 10 min, RT). The pellet was resuspended and counted. Cells were plated in a T25 flask (Nunc easy Flask) in DMEM low glucose, penicillin/streptomycin (Sigma Aldrich), L‑glutamine (Gibco) and 10% human AB-serum (German Red Cross Blood Donor Service). Medium was changed bi-weekly until bASC reached a subconfluent stage (approximately 75%). Cells were then split using trypsin/EDTA. Cells were counted and aliquots cryopreserved in fetal bovine serum (FBS)/10% DMSO (dimethyl sulfoxide, WAK-Chemie). Remaining cells were seeded as passage 1 (p1) cells at 200 cells/cm^2^. ASC growth rate was monitored recording cell number at every passage by calculating cell doublings (CD) and doubling time (DT):

$$cell\,\textit{doublings}\,\left(CD\right)=\frac{Log10\,\left(Fcn\right)-Log10\left(Icn\right)}{Log10\left(2\right)}\textit{doubling}\,time\left(DT\right)=\frac{\textit{Culture}\,\textit{duration}\,\left(h\right)}{CD}$$

where Fcn is final cell number and Icn the initial cell number.

To define multipotent MSCs, the International Society for Cell and Gene Therapy has suggested a characteristic combination of functional properties and the specific surface marker phenotype. The hallmarks include plastic adherence, trilineage differentiation capability into adipocytes, chondrocytes and osteoblasts as well as the characteristic surface molecular marker expression, defined by the absence of hematopoietic markers and the presence of CD73, CD90, and CD105 (14,15). After p1, cells were subjected to differentiation assays. Immunophenotyping was performed after p2. Growth curves were calculated in p1 and p2.

### Differentiation assays

Osteogenic and adipogenic differentiation was performed as described previously [7] using osteogenic and adipogenic induction/maintenance medium (Lonza). Briefly, after 21 days of induction, cells were stained with Oil Red O and von Kossa stain as described.

### Immunophenotyping

Multicolor immunophenotyping was also performed as described before [7]. Briefly, cells were trypsinized using trypsin/EDTA (Pan Biotech) and adjusted to 1 × 10^5^ cells/tube. FcR blocking reagent (Miltenyi Biotech) was added and incubated for 10 min before adding the titrated volume of antibody (see table S1). Unstained cells served as negative control. Cells were incubated 20 min, followed by two washes with PBS. Finally, Sytox Blue was added to exclude dead cells and cells were analyzed using a FACS Canto II analyzer running FACS Diva software (Becton Dickinson).

### Statistical analysis

Analysis was performed using GraphPad Prism 7. Significance testing was done using two-way analysis of variance (ANOVA) or *t*-test, as applicable.

## Results

### Isolation success rate, phenotype

**Fig. 1:**
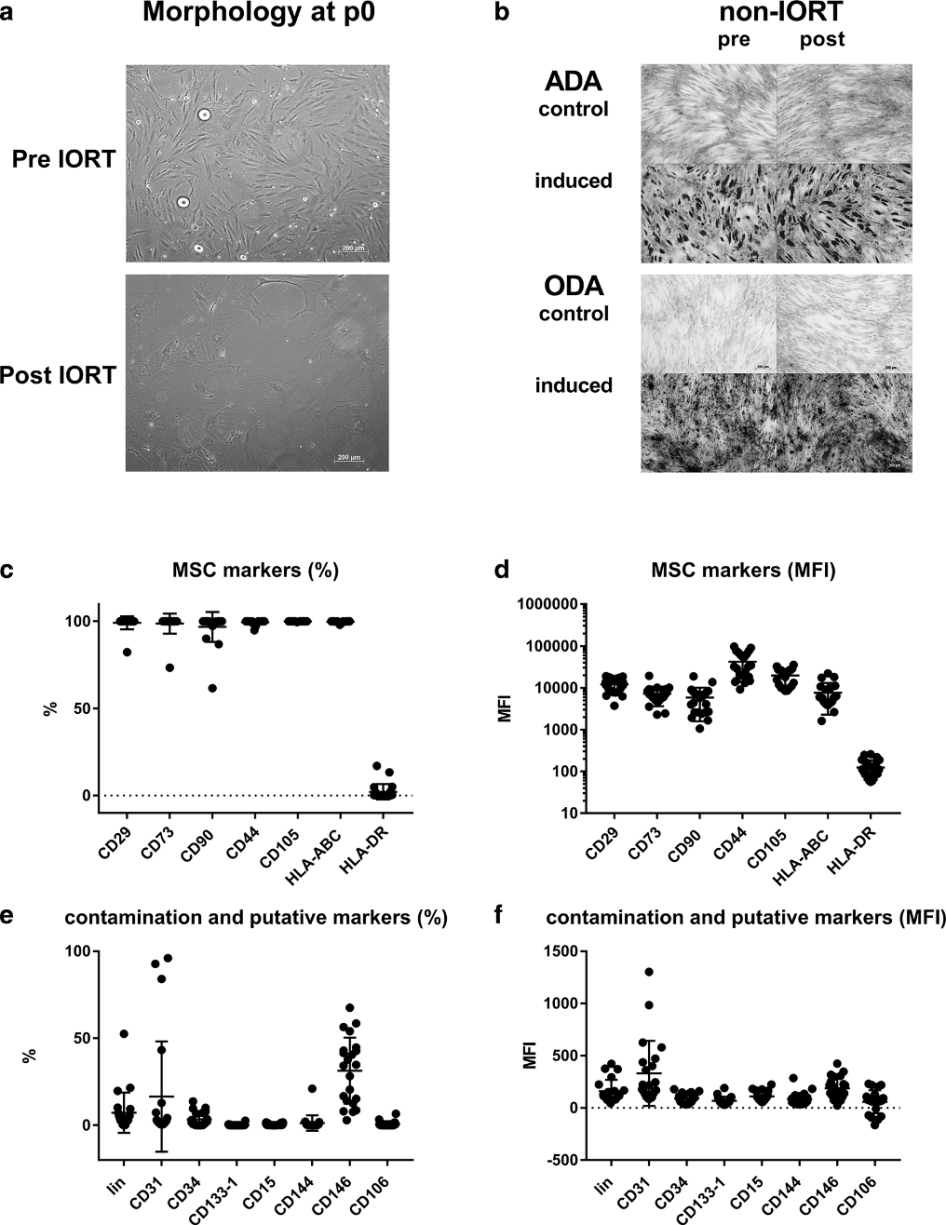
Characterization of breast-derived adipose stromal cells (bASC).** a** Phase contrast photomicrographs in p0 of a pre and post IORT sample. The typical mesenchymal stromal cells(MSC)-like morphology is apparent in the pre intraoperative radiotherapy (IORT) sample. All cells attaching from the post IORT sample show a senescent phenotype: cells lose their fibroblastoid shape, become flat and huge with bundles of stress fibers. To allow for better comparison, contrast and brightness were slightly adjusted. **b** Exemplary figures of adipogeneic (ADA, Oil Red O stain) and osteogenic (ODA, von Kossa stain) differentiation results displaying the negative controls (*top rows*) and the adipogenic/osteogenic-differentiated samples (*bottom rows*). **c**, **d** Flow cytometric assessment of MSC markers and **e**, **f** markers indicating contamination and other markers used to characterize putative subpopulations of MSC. Data are expressed as percent positive cells or mean fluorescence values MFI (median FL-A). No differences were seen; thus data are merged from all conditions. *MFI* mean fluorescence intensity, *ADA* adipogenic differntiation assay, *ODA* osteogneic differentiation assay

MSCs were isolated using collagenase digestion from breast tissue biopsies pre and post IORT (controls: pre and post sentinel lymph node resection). The sample weight was comparable with 0.45 ± 0.37 for pre non-IORT, 0.37 ± 0.26 post non-IORT and 0.56 ± 0.44 and 0.54 ± 0.29 g for pre and post IORT, respectively. In all, 95% of the pre IORT samples and 57% and 66% of the pre and post non-IORT samples yielded MSC cultures, growing beyond passage 2 (Table 1). However, adherent cells were observed in only one of the 20 samples post IORT. These, however, stopped proliferation already after a few days, reminiscent of a highly senescent phenotype marked by numerous stress fibers (Fig. 1a; [7]). In all other cultures, bASC in early in culture grew out as colonies forming cell monolayers with a typical fibroblastoid phenotype (Fig. 1a). The proliferation potential of non-IORT bASC, both pre and post, was comparable to ASC isolated from subcutaneous fat [7]. bASC from pre IORT samples proliferated significantly slower (Table 2). bASC from two exemplary cultures could be cultured up to 6–7 passages until reaching replicative senescence, without an indication of excessive or prolonged proliferation indicative of malignant transformation or tumorigenic origin (not shown).

**Table 1:** Isolation success in percent

	Non-IORT (*n* = 21)	IORT (*n* = 20)
Pre	12 (57%)	19 (95%)
Post	14 (66%)	1 (5%) no prolonged proliferation

Number and percentage of samples where bASC (breast adipos stromal cells) were isolated

*IORT* intraoperative radiotherapy

**Table 2:** Growth curves

	Pre Non-IORT		Post Non-IORT		Pre IORT	
	CD	DT	CD	DT	CD	DT
P2	5.57 ± 0.96	37.42 ± 13.04	5.44 ± 1.15	32.29 ± 6.25	4.51 ± 1.30	53.03 ± 19.74*
P3	5.68 ± 0.49	34.30 ± 6.46	5.85 ± 0.44	32.55 ± 6.99	5.1 ± 0.40	48.20 ± 10.14**

Cell doublings (CD) and doubling time (DT, in hours) are listed

*IORT* intraoperative radiotherapy

*DT p2: Post Non-IORT vs. pre-IORT *p* = 0.0355

**DT p3: Pre and post Non-IORT to Pre-IORT *p* = 0.0225 and *p* = 0.0119, respectively

### Differentiation assay

Differentiation assays and immunophenotyping were performed to verify the MSC-like nature of bASCs. Nearly 90% of the presamples underwent adipogenic differentiation, whereas from the post-non-IORT samples only 55% showed adipogenic differentiation properties (Table 3 and Fig. 1b). All bASC presamples differentiated into the osteogenic lineage, whereas this proportion again was reduced to 77% in the post non-IORT samples (Table 3 and Fig. 1b).

**Table 3:** Differentiation potential

	Pre Non-IORT	Post Non-IORT	Pre IORT
Adipogenic	88.89%	54.55%	87.5%
Osteogenic	100%	76.92%	100%

Percentage of samples that underwent adipogenic or osteogenic differentiation upon inductive medium treatment

*IORT* intraoperative radiotherapy

### Immune phenotype

The immune phenotype of bASC at p2 was highly typical for ASC and corresponded to the ISCT proposed positive and negative markers to distinguish MSCs from other cells [12, 13]. As expected, no difference in the marker expression of bASC was observed comparing pre/post non-IORT and pre IORT samples. Thus, all data were merged. Furthermore, cells expressed/non-expressed the typical MSC markers (Fig. 1c, d) at comparable intensities in all but one sample. Negativity of HLA class II indicates a nonactivated state of isolated bASC. Markers to control for contamination (such as lineage for hematopoietic cells) were not detected in the majority (Fig. 1e, f). Surprisingly, few samples contained a high proportion of CD31-postive cells, indicating an endothelial trait despite their fibroblastoid morphology. Other endothelial markers such as CD144 (VE-Cadherin) and CD106 (VCAM), however, were not expressed. In p2, CD34 was not expressed, although ASC may express CD34 early after isolation [13]. The CD146+ subset has been suggested to represent a population transitional between adipose stromal cells and pericytes. In fact, percentage of expressing cells varied markedly. Mean fluorescence intensity on positive cells, however, was highly comparable.

## Discussion

Our results indicate that IORT entirely abolishes the adhesion and proliferation potential of bASC, suggesting radiosensitivity of bASC, at least in situ. Since IORT is performed with 20 Gy prescribed to the applicator surface in a single session, supporting data that doses higher than 10 Gy radiosensitize MSC [9, 14]. The morphology of the one post IORT sample, in which few cells attached and proliferated, resembled a senescent phenotype [7]. Alessio et al. described a senescent MSC phenotype induced by radiation ranging from 40 mGy to 2 Gy in line with a loss of clonogenic capacity [15]. They showed that the few surviving clones, however, retained differentiation capacity fitting to previously published data suggesting a radioresistant subpopulation [14].

Despite the nearly high isolation success rate, pre IORT samples showed decelerated proliferation compared to non-IORT controls. Different subtypes or molecular phenotypes between the study and control cohort cannot explain these differences between pre IORT and pre/post non-IORT. The lower mean breast size in non-IORT women (exclusion criterion for IORT) and the likely different breast composition with a higher proportion of connective tissue, which was often observed in non-IORT samples, may explain the differing success rates. Although we tried to avoid thermocoagulation during biopsy collection, it is inevitable during breast-conserving surgery depending of the degree of bleeding. Nevertheless, within the groups there was no difference, so that we consider this effect as negligible.

Our data on osteogenic and adipogenic differentiation potential suggest that within the 30 min period after resecting the tumor and closing, the wound affects the differentiation—but not the outgrowth and proliferation—properties of bASC. Possibly the surgical stress induced (pro)inflammatory priming may has affected differentiation, previously shown to affect the balance between osteo- and adipogenic differentiation [14]. At the present stage, we cannot exclude that the extended stress caused by surgical tumor resection and IORT irradiation and subsequent biopsy affected bASC outgrowth rather than the IORT itself. We regard it important that the entire IORT procedure is effective in targeting the tumor niche.

Growth, morphology, differentiation, and immune phenotypic characterization documented that bASC fulfilled criteria of MSCs. IORT ultimately abolished the outgrowth capacity of bASC, indicating bASC radiosensitivity in situ. These data support the notion that IORT exerts an ablative effect within the tumor bed, not only affecting potential residual tumor cells, but also the tumor bed. By this, the IORT concept of localized radiation targeting residual tumor cells in the tumor bed to reduce the risk of relapse appears to be validated with respect to tumor bed-derived bASC. Our data may aid in explaining the efficiency of IORT concerning local recurrences and overall survival as stated in the follow-up of the TARGIT A trial [16]. They may further argue towards a reduced risk of breast cancer metastasis [17].

It was beyond the scope of this study to assess the effect of radiation of expanded MSC [9, 11, 14] and to investigate the potential impact of ionizing radiation on de novo recruitment of circulating MSC to the tumor bed as previously suggested [18, 19]. The question, if and how IORT may affect the recruitment of cells to the tumor bed and its local microenvironment, offers potential for future investigations.

## Conclusion

After breast-conserving surgery, approximately 90% of local recurrences emerge in close proximity to the initial tumor bed, potentially reflecting a significant contribution of the tumor bed to relapse. The goal of intraoperative radiotherapy (IORT) is to target remaining tumor cells by a locally concentrated dose of radiation while preserving healthy tissue. Our results indicate that IORT, besides the proven effect on breast cancer cells, entirely abolishes the adhesion and proliferation potential of bASC, suggesting radiosensitivity of bASC at doses of 20 Gy. This might add to reduce the risk of tumor relapse and metastases.

## Caption Electronic Supplementary Material

Table S1. Antibodies used for multicolor immunophenotyping

## Abbreviations

ASC: Adipose stromal cells
bASC: Breast-derived adipose stromal cells
DMEM: Dulbecco’s Modified Eagle Medium
DMSO: Dimethyl sulfoxide
EDTA: Ethylenediaminetetraacetic acid
EMT: Epithelial mesenchymal transition
FBS: Fetal bovine serum
Gy: Gray
IORT: Intraoperative radiotherapy
ISCT: International Society for Cell & Gene Therapy
min: Minutes
ml: Milliliters
MSC: Mesenchymal stromal cells
PBS: Phosphate-buffered saline
RT: Room temperature
SNB: Sentinel node biopsy
