# Supplementary material for: Intraoperative radiotherapy for breast cancer treatment efficiently targets the tumor bed preventing breast adipose stromal cell outgrowth
Source: Strahlenther Onkol. 2020 Feb 6;196(4):398–404. doi: 10.1007/s00066-020-01586-z (PMC7089893; doi:10.1007/s00066-020-01586-z)
Supplement: Supplementary file 1 — Table S1. Antibodies used for multicolor immunophenotyping [file 66_2020_1586_MOESM1_ESM.docx]

Table S1. Antibodies used for multicolour immunophenotyping

|  |  | Clone | Manufacturer | Order Number |
| --- | --- | --- | --- | --- |
| **0** | unstained | - | - | - |
| **1** | CD29-AlexaFluor 488  CD73-PE  CD90-APC | TS2/16  AD2  5E10 | BioLegend  BD  BD | 303016  550257  559869 |
| **2** | CD106-FITC  CD146-PE  CD44-APC | VCAM-1  TEA1/34  IM7 | BD  Beckman Coulter  BioLegend | 551146  A07483  103012 |
| **3** | CD3-FITC  CD14-FITC  CD235a-FITC  CD19-FITC  CD34-PE  CD45-FITC  CD105-APC | UCHT1  M5E2  GA-R2  HIB19  8G12  HI30  SN6 | BioLegend  BD  BD  BD  BD  BD  eBioscience | 300406  555397  559943  555412  345802  555482  17-1057-42 |
| **4** | CD15-FITC  CD31-APC | HI98  WM59 | BD  eBioscience | 555401  17-0319-73 |
| **5** | HLA-DR-FITC  CD144-PE  HLA-ABC-APC | L243  TEA1/31  G46-2.6 | BD  Beckman Coulter  BD | 555811  A07481  555555 |
